# Effectiveness of patient triage at the orthopedic hospital and the hygiene concept in a professional handball team in the first year of the SARS-CoV-2 pandemic

**DOI:** 10.1007/s00132-023-04358-6

**Published:** 2023-04-24

**Authors:** Pavel Varganov, Christian Riediger, Christoph Lohmann, Sebastian Illiger

**Affiliations:** Department of Orthopedics, University Hospital Magdeburg, House 8, Leipziger Str. 44, 39120 Magdeburg, Germany

**Keywords:** COVID-19, Sport, Handball, Outbreak, COVID-19, Sport, Handball, Ausbruch

## Abstract

The first severe acute respiratory syndrome coronavirus type 2 (SARS-CoV‑2) pandemic wave in Germany in spring 2020 challenged the largely unprepared healthcare system. A prevention concept was implemented to protect the vulnerable patient group at our orthopedic department. The patient triage during the pre-admission process included screening for symptoms and obtaining information on travel, occupation, contact and cluster (TOCC) [16].

In March 2020, all sporting events were also cancelled or postponed [12]. Mitigation strategies for sport activities were necessary to restart training and competition. For the professional handball team of the Sport Club Magdeburg (SCM), a hygiene concept including strict mitigation measures combined with a polymerase chain reaction (PCR) test regime was implemented.

We reviewed 15,739 patient contacts in a 12-month period at orthopedic department during the SARS-CoV‑2 pandemic. This epidemiological, retrospective study presents the results of patient triage detecting cases with suspected SARS-CoV‑2 infections when entering the clinic. We also considered 2328 inpatient PCR test results and the infection rates among the medical staff. At the same period, professional athletes underwent 1428 PCR tests as a part of the hygiene concept.

During the triage process, 333 cases (2.12%) with suspected SARS-CoV‑2 infections were detected at the orthopedic outpatient department. Three patients had a positive PCR test result after triage. Another four positive PCR tests were found among the inpatient group and one positive result among the medical staff. In the athletes’ cohort, none of the 1428 PCR tests was positive.

Patient triage as a part of the preadmission process is an effective tool to protect the maximum-care hospital from a SARS-CoV‑2 mass outbreak. A hygiene concept with a defined PCR test regime protects a professional athlete team from SARS-CoV‑2 infections during international competition and training.

**Graphic abstract:**

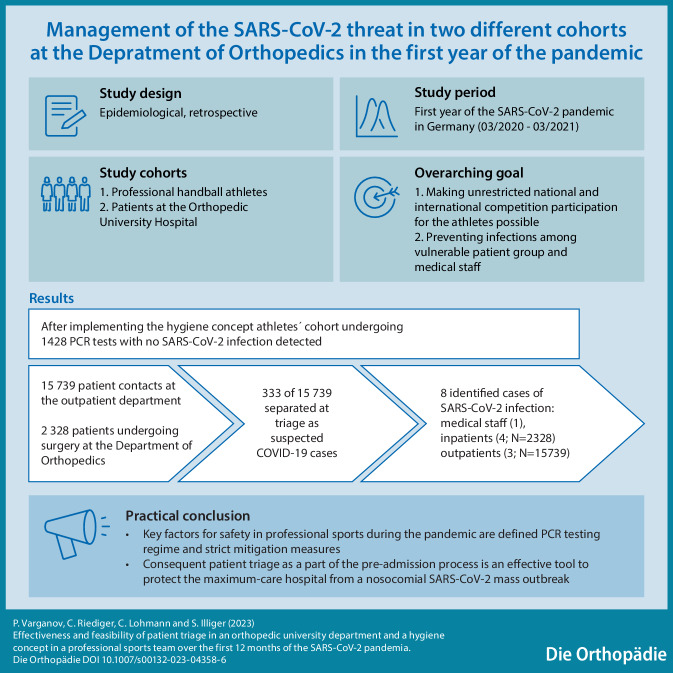

## Abstract (de)

Zum Schutz der meist unvorbereiteten medizinischen Einrichtungen vor Infektionsausbrüchen werden zu Beginn der SARS-CoV-2-Pandemie Präventionskonzepte implementiert. Eine Patiententriage beinhaltet die Erfassung der TOCC-Informationen (travel, occupation, contact, cluster). Dazu gehören Angaben über den Aufenthalt im Ausland oder Risikogebiet, Symptome sowie mögliche Kontakte zu einer infizierten Person (Stand 05/2020) [16]. Die Einschränkungen der Pandemie betreffen auch den Leistungssport [12]. Um Training und Wettkampf zu ermöglichen, wird für die Profi-Handballer des Sportclubs Magdeburg (SCM) ein Hygienekonzept mit PCR-Testregime erarbeitet.

Im Rahmen dieser epidemiologischen, retrospektiven Studie werden 15739 Patientenkontakte in der Orthopädischen Universitätsklinik im ersten Pandemiejahr ausgewertet. Zur Beurteilung der Effektivität der Patiententriage werden 2328 PCR-Testergebnisse stationärer Fälle sowie das Infektionsgeschehen unter dem medizinischen Personal mitberücksichtigt. In dem Untersuchungszeitraum unterziehen sich die Profi-Handballer 1428 PCR-Tests. In unserer Ambulanz konnten im Rahmen der Triage 333 (2.12 %) Verdachtsfälle identifiziert werden. Nach der Triage hatten drei ambulante und vier stationäre Patienten ein positives PCR-Testergebnis. Ein positiver Fall konnte unter dem medizinischen Personal festgestellt werden. Durch konsequente Umsetzung der Patiententriage sowie des Hygienekonzeptes für das Fachpersonal konnte ein unkontrollierter nosokomialer SARS-CoV-2-Ausbruch verhindert werden. Durch striktes Einhalten der Hygienevorschriften zusammen mit engmaschiger PCR-Testung konnte bei voller nationaler und internationaler Wettbewerbsbeteiligung kein SARS-CoV-2-Fall bei den Sportlern des SCM detektiert werden.

The SARS-CoV-2 pandemic and its impact on society have been one of the key topics in public discourse since the outbreak in 2019, involving research groups from various fields of activity.

The current paper examines the effects of the pandemic on professional sports as well as the sphere of medical care, using an orthopedic hospital and a professional sports team as examples and assessing the effectiveness of the pandemic prevention strategies used in the two aforementioned areas.

## Introduction and background

In Germany, the first severe acute respiratory syndrome coronavirus 2 (SARS-CoV-2) patient was isolated in a clinic in Munich on 27 January 2020 [5]. A few weeks later, all German hospitals reduced their capacities in the sector of elective surgery by up to 49% [1, 19]. Orthopedic surgery was one of the most affected branches. The number of total knee arthroplasty operations decreased by 80% in April 2020 [1]. During the first pandemic wave in spring 2020 there were neither vaccines against the virus nor specific treatment options for the coronavirus disease 2019 (COVID-19). For the vulnerable group of patients (immunocompromised tumor patients, patients with rheumatic disease, older multimorbid patients etc.) visiting clinic meant a significant risk of infection. Also, a higher mortality rate among COVID-19 patients undergoing elective surgery [13] made the protection of the orthopedic ward from virus outbreak a prioritized task. To proceed with the treatment of orthopedic patients and to protect the vulnerable group, mitigation strategies were necessary. In the outpatient clinic of the orthopedic department a standardized triage procedure was implemented to identify cases with suspected SARS-CoV‑2 infections.

In March 2020 all sporting events and competitions were called off or postponed due to the first national lockdown [10]. To restart the activities, also in professional sports, hygiene concepts had to be established. For the professional handball team of the Sport Club Magdeburg (SCM), such a concept was developed in cooperation with the Department of Orthopedics at the University Hospital Magdeburg and implemented in August 2020. The hygiene concept included strict mitigation measures and a PCR test regime to protect the team and the staff taking part in national and international matches.

## Study design and investigation methods

### Triage at the department of orthopedics.

**Fig. 1:**
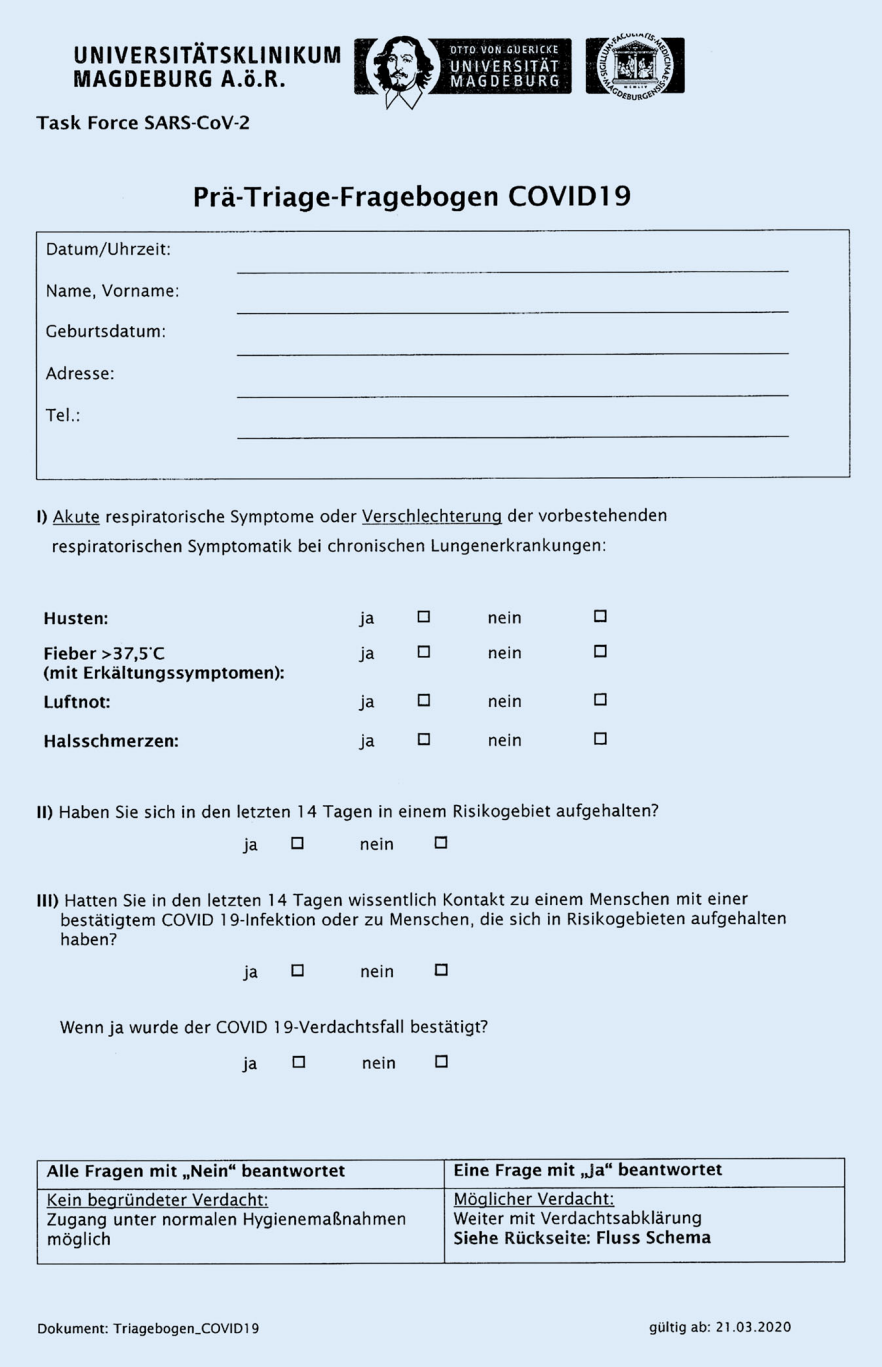
Pretriage questionnaire (status March 2020, University Hospital Magdeburg)

At the beginning of the SARS-CoV‑2 pandemic in 2020, the University Hospital Magdeburg and the Department of Orthopedics implemented patient triage systems to identify patients with suspected SARS-CoV‑2 infections. At the campus entrance non-medical staff questioned patients and visitors using the pretriage questionnaire (Fig. 1).

Persons who demonstrated acute respiratory symptoms (or exacerbation of chronic disease, such as cough, fever, shortness of breathing, sore throat), travelled to COVID-19 hot spot regions in the last 14 days or contacted people known to be infected with SARS-CoV‑2 were categorized as suspected COVID-19 patients. This group was redirected to the fever center. All negative cases were allowed to proceed to their destination clinics.

**Fig. 2:**
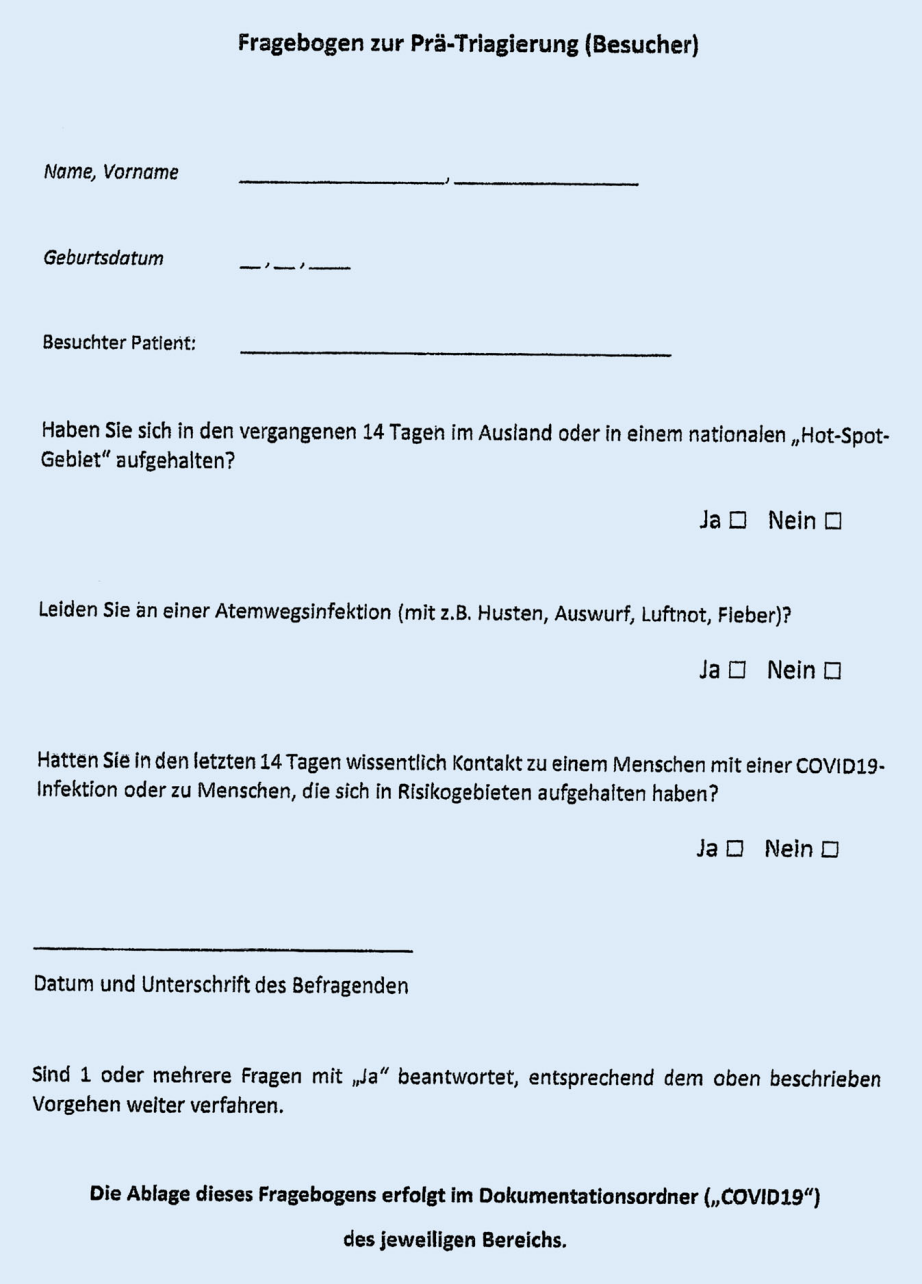
Triage questionnaire Department of Orthopedics (status March 2020, University Hospital Magdeburg)

A second triage system in the outpatient clinic at the Department of Orthopedics involved medical staff using a similar triage questionnaire (Fig. 2) and collecting information about residence in national hot spots or abroad during the last 14 days, acute COVID-19 symptoms and contacts with SARS-CoV‑2 infected cases. The selection of negative cases at the first triage led to a low number of positive cases at the second triage. Non-contact temperature measurement with a cut-off value of 37.5 °C, according to the WHO recommendation [3], was a part of the triage process. During the second pandemic wave in autumn 2020 and increasing infection rates, especially national hot spots had to be considered during triage process. The districts our patients were coming from were noted down on the triage questionnaire beginning with calendar week number 45 (2 November 2020). Districts with 7‑day incidence rates over 200 per 100,000 population were defined as national hot spots.

We reviewed 15,739 triage questionnaires of our patients and visitors in the orthopedic outpatient department of the Orthopedic University Clinic Magdeburg in a 12-month period from 1 March 2020 to 28 February 2021. Persons categorized as suspected COVID-19 cases could enter the facility only in cases of emergency. All symptomatic cases were redirected to the fever center or general practitioner. Persons travelling abroad during the last 14 days were allowed to enter the clinic with a negative PCR test result not older than 48 hours. For patients who contacted SARS-CoV‑2 infected persons, a 14-day quarantine was required, based on the official health department´s recommendation. After the self-quarantine period a negative PCR test result not older than 48 hours had to be presented at the triage.

We also reviewed PCR test results of suspected COVID-19 cases among orthopedic patients which were performed in our outpatient department, orthopedic ward and fever center. Part of the suspected cases were tested externally after the triage and not at the University Hospital Magdeburg.

### Hygiene concept of the SCM.

The hygiene concept for the SCM handball players included logistical and organizational measures to reduce virus transmission rate. Three central strategies were implemented at the sport facilities during training and matches: splitting up the involved persons in active and passive participants; defining zones (court, locker room, grandstand); consequent spatial and time wise separation of the groups [8]. Staff members with high risk for developing severe COVID-19 disease in cases of infection have been identified during medical checks and protected through additional measures [8]. The hygiene concept also included a PCR test regime according to the current regional 7‑day incidence rate [8]. We performed 1428 PCR tests of the 25 team members at our orthopedic outpatient department during the study period.

## Results

### Triage at the Department of Orthopedics.

A total of 333 cases out of 15,739 were separated at triage as suspected COVID-19 cases (2.12%).

The number of patients coming from national hot spots with 7‑day incidence rates over 200 per 100,000 population in the county was 229 (69%).

**Fig. 3:**
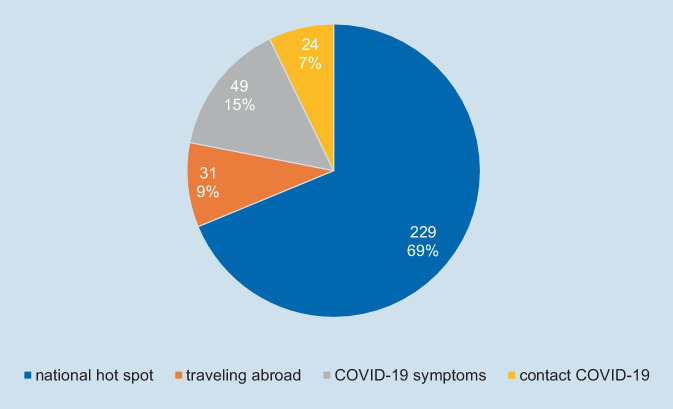
Positively triaged cases at the orthopedic outpatient department (16 March 2020–28 February 2021)

Another 49 patients (15%) demonstrated COVID-19 symptoms at the triage, 31 persons (9%) had travelled abroad during the last 14 days, and 24 persons (7%) contacted people known to be infected with SARS-CoV-2 during the last 14 days (Fig. 3).

**Fig. 4:**
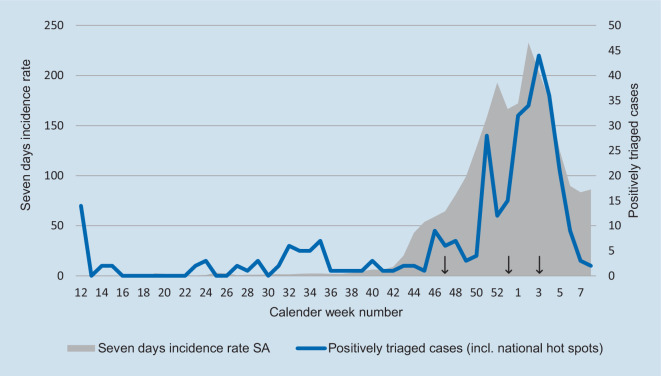
Triage results at the orthopedic outpatient department related to 7‑day incidence rate in the federal state Saxony Anhalt (16.03.2020–28.02.2021). Three cases of SARS-CoV‑2 infection detected during calendar week number 47, 53 and 3 (*arrow*)

We reviewed 15,739 contacts with patients and visitors in a 12-month period from 1 March 2020 to 28 February 2021, during the SARS-CoV‑2 pandemic at the orthopedic outpatient department of the Orthopedic University Hospital Magdeburg. Beginning with calendar week number 45 (2 November 2020), the districts our patients were coming from were noted down on the triage questionnaire. Our patients arrived from 45 different districts. The 7‑day incidence rates of these districts were researched using data provided by the Robert Koch Institute (corona-in-zahlen.de; health-mapping.de). In 33 of these 45 districts, the incidence rates increased during the second pandemic wave in winter 2020 to over the 200 mark for 38 days on average. Three PCR positive cases of SARS-CoV‑2 infection (0.02%) were detected during calender week number 47, 53 and 3 (Fig. 4).

In the study period, there was one single laboratory-confirmed COVID-19 case among medical staff, whereby the infection was related to household exposure.

There were 2328 patients undergoing surgery at the Departments of Orthopedics in the study period. Routine PCR tests during the admission process identified four cases of SARS-CoV‑2 infection (0.2%). Only two of these cases had to be isolated on the peripheral COVID unit demonstrating mild COVID-19 symptoms.

### Hygiene concept of the SCM.

Despite the very high logistical effort, the hygiene concept could be implemented with a large number of highly cost-intensive PCR tests for the athletes. During the study period (1 March 2020–28 February 2021) and after implementing the hygiene concept in August 2020, we performed 1428 PCR tests at the orthopedic outpatient department. Due to strict compliance with the hygiene regulations combined with flexible PCR test regime related to current incidence rates, no SARS-CoV‑2 case could be detected among the athletes of SC Magdeburg, making unrestricted national and international competition participation possible (Table 1).

**Table 1:** PCR test results among SCM handball players (1 March 2020–28 February 2021)

	Number of athletes *N* = 25
Number of PCR tests performed among athletes	1428
Number of positive PCR test results	*0*

*PCR* polymerase chain reaction, *SCM* Sport Club Magdeburg

## Discussion and conclusion

The Corona virus was responsible for two pandemics during the 21st century [4]. The SARS-CoV‑1 pandemic caused in November 2003 more than 8000 infections with a high mortality rate of nearly 10% [14, 18]. Infection rates among health care workers of up to 30% were also very high [14]. There were neither vaccines against the virus nor specific treatment for severe acute respiratory syndrome (SARS), with its incompletely known pathogenesis [18]. The German health care system was confronted with comparable circumstances at the beginning of the SARS-CoV‑2 pandemic in March 2020. Infection rates among health care workers were three times higher than the federal average [22, 25]. The Robert Koch Institute informed weekly about the increasing number of SARS-CoV‑2 outbreaks in German hospitals [20]. Significant higher mortality rates were reported for patients undergoing emergency and elective surgery [6, 7, 13, 17]. In the absence of vaccines and specific treatment for the COVID-19 disease, preventive strategies had to be implemented to protect our patients, especially the vulnerable groups. Patient triage, together with a specific testing strategy, increased patient safety and prevented a SARS-CoV‑2 outbreak in orthopedic department of a maximum-care hospital. During the study period, 8 cases of SARS-CoV‑2 infection in total were detected among: medical staff (1), inpatients (4; *N* = 2328) and outpatients (3; *N* = 15,739). Patient triage by medical staff before entering the clinic can be an effective tool for preventing mass infection.

Up to 35% of German and Austrian professional athletes were quarantined and 0.5% hospitalized during the first pandemic year in Germany [2]. Despite the fact that professional athletes rarely develop severe clinical COVID-19 symptoms [9, 15, 21], a severe illness can lead to longer rehabilitation and affect eligibility [21]. Key factors for safety during professional sports events are the reduction of transmission risk by strict mitigation measures and the identification of positive cases by regular PCR testing [11, 23, 24]. To restart training and competition for the professional athletes of the Sport Club Magdeburg during the SARS-CoV‑2 pandemic, a hygiene concept was implemented in August 2020. The PCR testing strategy, as an important part of this concept, was realized by the medical staff of our outpatient department with 1428 PCR tests for 25 team members during the first and second pandemic waves in Germany (March 2020–February 2021). The PCR test regime referring to a 7‑day incidence rate together with strict hygiene concept protected the athletes from any SARS-CoV‑2 infection during the study period.

## Practical conclusion

- Consistent patient triage as a part of the preadmission process is an effective tool to protect the maximum-care hospital from a nosocomial severe acute respiratory syndrome coronavirus type 2 (SARS-CoV‑2) mass outbreak.
- Key factors for safety in professional sports during the pandemic are the reduction of virus transmission risk by strict mitigation measures and the identification of positive cases by regular polymerase chain reaction (PCR) testing, referring to the 7‑day incidence rate.

## Declarations

All procedures followed were in accordance with the ethical standards and the statement of the appropriate ethics committee at the University Hospital Magdeburg. This article does not contain any studies with animal subjects. We confirm that this manuscript has not been published elsewhere and is not under consideration by another journal. All authors have approved the manuscript and agree with its submission to *Die Orthopädie*.
